# Role of Mitochondrial Reactive Oxygen Species-Mediated Chaperone-Mediated Autophagy and Lipophagy in Baicalin and N-Acetylcysteine Mitigation of Cadmium-Induced Lipid Accumulation in Liver

**DOI:** 10.3390/antiox13010115

**Published:** 2024-01-17

**Authors:** Jian Sun, Yan Chen, Tao Wang, Waseem Ali, Yonggang Ma, Zongping Liu, Hui Zou

**Affiliations:** 1 College of Veterinary Medicine, Yangzhou University, Yangzhou 225009, China; dz120200001@stu.yzu.edu.cn (J.S.); mx120211002@stu.yzu.edu.cn (Y.C.); dx120200175@stu.yzu.edu.cn (T.W.); 008301@yzu.edu.cn (W.A.); 007854@yzu.edu.cn (Y.M.); liuzongping@yzu.edu.cn (Z.L.); 2 Joint International Research Laboratory of Agriculture and Agri-Product Safety, The Ministry of Education of China, Yangzhou University, Yangzhou 225009, China; 3 Jiangsu Co-Innovation Center for Prevention and Control of Important Animal Infectious Diseases and Zoonoses, Yangzhou 225009, China

**Keywords:** baicalin, N-acetylcysteine, mitochondrial ROS, chaperone-mediated autophagy, lipophagy

## Abstract

Cadmium (Cd) is a major health concern globally and can accumulate and cause damage in the liver for which there is no approved treatment. Baicalin and N-acetylcysteine (NAC) have been found to have protective effects against a variety of liver injuries, but it is not clear whether their combined use is effective in preventing and treating Cd-induced lipid accumulation. The study found that Cd increased the production of mitochondrial reactive oxygen species (mROS) and elevated the level of chaperone-mediated autophagy (CMA). Interestingly, mROS-mediated CMA exacerbates the Cd-induced inhibition of lipophagy. Baicalin and NAC counteracted inhibition of lipophagy by attenuating Cd-induced CMA, suggesting an interplay between CMA elevation, mitochondrial destruction, and mROS formation. Maintaining the stability of mitochondrial structure and function is essential for alleviating Cd-induced lipid accumulation in the liver. Choline is an essential component of the mitochondrial membrane and is responsible for maintaining its structure and function. Mitochondrial transcriptional factor A (TFAM) is involved in mitochondrial DNA transcriptional activation and replication. Our study revealed that the combination of baicalin and NAC can regulate choline metabolism through TFAM and thereby maintain mitochondrial structure and functionality. In summary, the combination of baicalin and NAC plays a more beneficial role in alleviating Cd-induced lipid accumulation than the drug alone, and the combination of baicalin and NAC can stabilize mitochondrial structure and function and inhibit mROS-mediated CMA through TFAM-choline, thereby promoting lipophagy to alleviate Cd-induced lipid accumulation.

## 1. Introduction

Cadmium (Cd) is a heavy metal commonly occurring alongside sphalerite. Consequently, the mining, smelting, and refining of zinc sulfide ore lead to the release of excess Cd into the environment [1]. Simultaneously, the widespread usage of Cd in industry and agriculture contributes to human activities that augment the levels of Cd pollution in soil, water, and air. These activities include waste discharge, waste incineration, and the application of fertilizers, all of which lead to an increase in Cd pollution within the food chain [2]. In non-occupational exposure populations, Cd exposure is predominantly from diet, soot, and tobacco. Food accounts for around 90% of Cd exposure in nonsmokers with cereals and vegetables usually being the main sources [3]. Cd is a cumulative toxicant with multiple targets, and the liver is its primary site of accumulation. This leads to the development of several liver diseases, such as non-alcoholic fatty liver disease (NAFLD), liver fibrosis, cirrhosis, and liver cancer [4]. Research has demonstrated that Cd exposure disrupts glucose regulation and hormonal balance [5]. Furthermore, the essential enzymes involved in carbohydrate metabolism, for example, glucose-6-phosphatase, displayed substantial alterations [6]. Metabolomic analysis revealed significant changes in various metabolic pathways (including glycolysis and gluconeogenesis, biopterin metabolism, tryptophan metabolism, tyrosine metabolism, glycerophospholipid metabolism, and fatty acid metabolism) in PC12 cells exposed to Cd [7]. However, the link between Cd-induced metabolic changes and lipid accumulation remains unknown.

According to increasing evidence, the accumulation of neutral lipids, such as triglycerides (TG) and cholesterol (TC), is one of the primary features of NAFLD [8]. The liver may experience excessive lipid buildup due to increased TG synthesis, decreased catabolism, or decreased very-low-density lipoprotein (VLDL) synthesis [9]. Epidemiological research has demonstrated that exposure to Cd heightens the likelihood of NAFLD [10], while investigations into American teenagers have shown that exposure to Cd during adolescence increases the probability of developing NAFLD in middle age [11]. Previous studies have shown that exposure to Cd leads to an increase in liver lipid accumulation by enhancing the fatty acid synthase (FAS)and Acetyl-CoA-Carboxylase (ACC)-mediated fatty acid production pathways [12]. Notably, Cd exposure has been found to increase intrahepatic cholesterol through HMG-CoA reductase (HMGCR) and improve TG transport by increasing VLDL as observed in the study conducted by Wenli Guo et al. [13]. Thus, it is suggested that there are variations in the effects of Cd on lipid production in different species. The impact of Cd on lipid degradation has remained debatable, with contrary outcomes observed in adipose and liver tissue. Cd enforces lipolysis in adipose tissue [14] and restrains it in liver [15]. The impact of Cd on the autophagy process of lipid droplets in the liver requires further investigation. It is crucial to determine the effect of Cd on the degradation of lipid droplets and its mechanism. Currently, there are no FDA-approved pharmaceuticals available for the treatment of Cd poisoning. Therefore, it is imperative to explore potential drugs that can efficiently mitigate Cd poisoning. Over the last twenty years, researchers have revealed that numerous natural compounds and their key active molecules (such as silymarin, ellagic acid, lutonin, rutin, and glycyrrhizic acid) have been utilized for the treatment of liver fibrosis, viral hepatitis, fatty liver, and cirrhosis [16,17]. Baicalin (5,6-dihydroxy-7-O-glucuronide) is a flavonoid primarily obtained from the root of S. baiclensis, which has been the subject of numerous in vitro and in vivo studies. These studies have demonstrated that baicalin has various pharmacological properties, such as antioxidant, antiviral, and anti-inflammatory effects. Additionally, baicalin has been demonstrated to assist in reducing obesity, improve liver function after injury, and alleviate alcohol-induced liver disease [18]. Baicalin has demonstrated efficacy as a combination strategy for chemotherapy adjuvants in various cancers and related signaling pathways [19]. Furthermore, baicalin is frequently utilized as an adjuvant treatment for hepatitis. A clinical trial revealed that a singular dose of baicalin (500 mg/kg) combined with cyclosporine A was safe and well-tolerated among adult participants, with no severe adverse effects reported [20]. N-acetylcysteine (NAC) is a medication and dietary supplement typically employed as a mucolytic agent to manage paracetamol overdose [21]. NAC can stimulate glutathione production and inhibit lipid peroxidation, regulating oxidative balance. Studies suggest that combining NAC with substances like lycopene and bromelain can lead to better therapeutic outcomes [22,23].

However, the potential of baicalin and NAC to mitigate Cd-induced lipid accumulation in the liver through lipid metabolism regulation and produce a synergistic effect remains uncertain. This study aims to evaluate the preventive and therapeutic capabilities of baicalin and NAC for Cd-induced lipid accumulation in vivo and in vitro.

## 2. Materials and Methods

### 2.1. Chemicals and Antibodies

Cadmium chloride (CdCl_2_, anhydrous, 439800) was purchased from Sigma-Aldrich (Carlsbad, CA, USA). AAV8-TFAM, TFAM overexpression (TFAM OE), PLD1 overexpression (PLD1 OE) plasmid was purchased from Genechem (Shanghai, China), Baicalin, MitoQ, and Choline chloride were obtained from MedChemExpress (Monmouth Junction, NJ, USA). N-acetylcysteine, Oil Red O Staining Kit, dihydroethidium (DHE), and Hoechst 33258 were purchased from the Beyotime Institute of Biotechnology (Shanghai, China). BODIPY™ 493/503 and MitoSOX™ were obtained from Thermo Fisher Scientific (Madison, WI, USA). The triglyceride (TG) assay kit, Ttotal cholesterol (TC) assay kit, malondialdehyde (MDA) assay kit, total antioxidant capacity (T-AOC) assay kit, superoxide dismutase (SOD) assay kit, reduced glutathione (GSH) assay kit, and catalase (CAT) assay kit were provided by Nanjing Jiancheng Bioengineering Institute (Nanjing, China). Primary antibodies against the following proteins were used: ACC, FAS, SCD1, LC3, SQSTM1/p62, β-actin, Perilipin, and PNPLA. These were purchased from Cell Signaling Technology (Danvers, MA, USA). LAMP2A was purchased from Abcam (Cambridge, UK). The secondary antibodies (Goat Anti-Mouse IgG(H+L) (115-035-003) and Goat Anti-Rabbit IgG (H+L) (111-035-003)) were purchased from Jackson ImmunoResearch (West Grove, PA, USA). HSPA8/HSC70 was purchased from Santa Cruz (Dallas, TX, USA). The TFAM, Choline and ophthalmic acid ELISA assay kit were purchased from Shanghai Enzyme-linked Biotechnology (Shanghai, China). The ATP Content Assay Kit, Micro Mitochondrial Respiratory Chain Complex IV, and V activity Assay Kit were provided by Solarbio (Beijing, China). The Cell Counting Kit-8 (CCK8) was provided by Yeasen BioTechnology (Shanghai, China).

### 2.2. Animals and Treatment 

Fifty male C57BL/6J mice (8 weeks old, weighing between 18–25 g) were procured from Cyagen Biosciences (Santa Clara, CA, USA). The mice were accommodated in a temperature-controlled room (24 ± 5 °C, 12 h light/dark cycle) with unrestricted access to food and water. The experimental protocols were approved by the Animal Care and Use Committee of Yangzhou University and were in compliance with the European Communities Council Directive 2010/63/EU for animal experiments. (approval ID: SYXK (Su). After acclimatizing for one week, the mice were divided randomly into five groups: a normal group (Control), a Cd exposure group (Cd), an NAC + Cd group, a Baicalin + Cd group, and a Baicalin + NAC + Cd group. In total, 20 mg/kg of baicalin and 10 mg/kg of NAC were given to mice in the Baicalin + Cd and NAC + Cd groups, respectively, while they were exposed to a 50 mg/L Cd solution. We prepared a solution of baicalin and NAC in normal saline at the mentioned concentration values with a fixed administration volume of 0.01 mL per gram of body weight, and we administered these agents continuously through oral gavage to all mice for 90 days.

Albumin (Alb)-cre mice were obtained from Cyagen Biosciences (Santa Clara, CA, USA) The AAV8-TFAM construct was injected through the tail vein, and the mice were subsequently housed in a suitable environment for one month to detect TFAM expression levels. The 32 mice were divided into four groups. Group 1 received AAV8-Negative Control (AAV8-NC), which was administered intraperitoneally (i.p.) with double-distilled water (DDW). Group 2 received AAV8-NC+Cd, which was administered i.p. with 1 mg/kg CdCl_2_. Group 3 received AAV8-TFAM, which was administered i.p. with DDW. Group 4 received AAV8-TFAM+Cd, which was administered i.p. with 1 mg/kg CdCl_2_. Intraperitoneal injections were given to all four groups for two weeks. The mice were fasted for 12 h prior to harvesting their tissues. Carbon dioxide asphyxiation was used as a humane method to kill the mice.

### 2.3. Cell Culture and Treatment

The alpha mouse liver 12 (AML12) cell line (AML12, ATCC^®^CRL-2254^TM^, Rockville, MD, USA) was cultured in Dulbecco’s modified Eagle’s medium (DMEM)/F-12 supplemented with 1% Insulin-Transferrin-Selenium-Sodium Pyruvate (ITS-A), 10% fetal bovine serum (FBS), 100 U/mL penicillin, and 100 mg/mL streptomycin, and maintained at 37 °C with 5% CO_2_. CdCl_2_ (ultrapure water—dissolved), separately stored at 4 °C, and diluted into working solutions before use. The experimental design was as follows: (1) Cells were treated with 5 μM Cd, 10 μM Baicalin, and 20 μM NAC alone or in combination, for 12 h to perform the subsequent assays; (2) Cells were treated with 5 μM Cd and 10 μM choline alone or in combination for 12 h to perform the subsequent assays; (3) Cells were transfected with PLD1 OE and/or treated with 5 μM Cd for another 12 h to perform the subsequent assays; (3) Cells were transfected with TFAM OE and/or treated with 5 μM Cd for another 12 h to perform the subsequent assays.

### 2.4. Transmission Electron Microscopy

Cells and liver samples were washed twice with PBS, then fixed in 2.5% glutaraldehyde for 2 h at room temperature before cooling at 4 °C for a further 12 h. Following fixation in osmic acid, the samples underwent dehydration, embedding, ultrathin slicing, and negative staining, followed by electron microscopic examination.

### 2.5. Superoxide Anion Detection

The excised liver sample underwent pretreatment and embedding in optimal cutting compound (OTC), then it was transformed to cryosections. The standard protocol incorporated treating with DHE dye for 20 min, followed by immersion in flowing tap water. Coverslips were sealed over these slices, which enabled fluorescence microscopy examination. Cells were treated with 5 μM Cd, 10 μM Baicalin, and 20 μM NAC alone or in combination, for 12 h, washed twice with PBS, and incubated with DHE dye at 37 °C for 20 min. The samples were observed under a fluorescence microscope.

### 2.6. Detection of Oxidative Stress-Related Indexes 

First, in accordance with the pre-experimental findings, a specific quantity of tissue was weighed to create a 2% tissue suspension. This was centrifuged at 1000× *g* for 5 min and the supernatant was collected for measurement. Superoxide dismutase (SOD) and catalase (CAT) activities were measured in the liver tissues using specific commercial kits from the Nanjing JianchengBioengineering Institute (Nanjing, China) according to the manufacturer’s instructions. The levels of glutathione (GSH) and malondialdehyde (MDA) in the liver tissues were measured with commercial kits from the Nanjing JianchengBioengineering Institute (Nanjing, China). Total Antioxidant Capacity (T-AOC) was measured in the liver tissues by specific commercial kits from the Nanjing JianchengBioengineering Institute (Nanjing, China) according to the manufacturer’s instructions.

### 2.7. Detection of Micro Mitochondrial Respiratory Chain Complex IV, V Activity and ATP Content

For fresh tissue samples, we first weighed out a specific amount of tissue and used a glass grinder to grind it 30 times. We then collected the liquid and centrifuged it, and then took the resulting supernatant for measurement. When dealing with cell samples, we used a cell scraper to scrape the cells from the dish and added them to a glass grinder. We ground the cells 25 times, collected the liquid, and centrifuged it. Finally, we removed the supernatant and take took it for measurement. Following the instructions provided by the manufacturer, we utilized an adequate quantity of supernatant to quantify the ATP content and enzyme activity of the IV and V mitochondrial respiratory chain complexes using a particular specific commercial kit (Solarbio, Beijing, China).

### 2.8. Oil RedO and BODIPY Staining

The frozen sections of liver were first covered with Oil Red O staining wash for 20 s, followed by immersion of the sections in the staining working solution and staining for 20 min. The staining solution was aspirated. The sections were covered with staining wash solution, incubated for 20 s, rinsed twice with PBS and observed under the microscope. BODIPY, a lipophilic fluorophore, marks polar lipids, particularly neutral lipid content in lipid droplets in cells. For cell samples, 4% paraformaldehyde fixation was carried out for 15 min, and the cells were washed twice with PBS for 2 min each time and then incubated with staining working solution for 20 min. The staining working solution was aspirated off, the cells were washed with PBS, and finally, Hoechst staining solution was added and incubated for 10 min. The cells were then washed with PBS three times before being imaged under the fluorescence microscope.

### 2.9. Western Blotting Analysis 

For tissue samples, the proteins were harvested from freshly dissected or preserved liver using lysis buffer. For cellular samples, cells grown on dishes were collected using a cell scraper, centrifuged, lysed in Radioimmunoprecipitation assay (RIPA) Lysis Buffer at 4 °C for 30 min, and treated with ultrasound twice for 5 s each time. Protein estimation utilized the Bradford protein assay (PC0010, Solarbio), followed by sodium dodecyl sulfate–polyacrylamide gel electrophoresis separation before subsequently being transferred onto polyvinylidene difluoride membranes (Millipore, Burlington, MA, USA). Immunoblotting proceeded post primary and secondary antibody incubations, using Image Lab software 2.0.1 (Bio-Rad, Hercules, CA, USA) for densitometric quantitation. Primary antibodies against the following proteins were used: ACC (C83B10), FAS (C20G5), SCD1 (C12H5), LC3 (2775), SQSTM1/p62(5114), β-actin (4967), Perilipin-2(95109), and PNPLA2(2138). These were purchased from Cell Signaling Technology (Danvers, MA, USA); LAMP2A (ab125068) was purchased from Abcam (Cambridge, UK). The second antibodies, namely Goat Anti-Mouse IgG (H+L) (115-035-003) and Goat Anti-Rabbit IgG (H+L) (111-035-003)) were purchased from Jackson ImmunoResearch (West Grove, PA, USA). HSPA8/HSC70(sc-7298) was purchased from Santa Cruz (Dallas, TX, USA).

### 2.10. Immunofluorescence (IF) Staining

AML12 cells seeded in 24-well plates were grown to about 60% confluence. After a series of processing, we removed the culture medium and washed the PBS twice. We then fixed the samples with PFA (4%, 10 min), permeabilized them with Triton X-100 (0.1%, 15 min), blocked them with BSA (2%, 90 min) at RT, and incubated them with the primary antibody (LAMP2A (1:100 dilution) and LC3 (1:100 dilution) mixture) or (HSC70 (1:100 dilution) and the BODIPY staining solution (1:1000 dilution) overnight at 4 °C. After being washed with PBS for three times, the cells were incubated with the secondary antibody (1:500 dilution) for 90 min and with DAPI for 5 min at RT, then the cells were observed under a confocal microscope. 

### 2.11. TG and TC Content Determination

The tissues were weighed then mixed with normal saline (1:9 ratio), then homogenized and centrifuged (2500× *g*, 10 min), retaining the supernatant for assaying. Cell samples were made into a cell suspension using normal saline, disrupted using ultrasound, centrifuged, and the supernatant was retained for measurement. The TG and TC contents in the supernatants were determined according to the protocol of the corresponding test kit.

### 2.12. Cell Viability Assay

The CCK8 assay kit was used to detect cell viability. First, the required CCK8 working solution (CCK8 solution and medium 1:9 configuration) was calculated according to the number of samples and protected from light. Cells were seeded in 96-well plates, and after a series of treatments, the medium was discarded and the CCK8 working solution was added. After incubation for 1 h, the OD value of each well was determined at 450 nm.

### 2.13. TFAM, Choline and Opthalmic Acid ELISA Test Kit

For tissue samples, a certain amount of fresh tissue was weighed and a tissue suspension was prepared for testing according to the preparation process. For cell samples, cells were first collected with pancreatic digestion, centrifugation, ultrasound, and cell suspension was prepared for testing. The standard and the sample to be measured were added to the enzyme-coated plate, the microplate reagent was added, sealed with a sealing film, and incubated at 37 °C for 60 min; the liquid was discarded, the washing solution was discarded after 30 min of the washing solution, and this was repeated 5 times, the chromogenic solution A and B were added, incubated at 37 °C in the dark for 15 min, the stop solution was added, and the absorbance was detected by the microplate reader.

### 2.14. Statistical Analysis

The data from at least three independent experiments were statistically analyzed and expressed as the mean ± standard deviation (SD). GraphPad Prism 6 software 6.07 (GraphPad Software Inc., La Jolla, CA USA) was used to analyze the data with one-way analysis of variance (ANOVA) (Scheffe’s SF test). *p* values less than 0.05 were considered to indicate significant differences.

## 3. Results

### 3.1. The Combination of Baicalin and NAC Protects Mitochondria and Reduces Oxidative Stress

**Figure 1:**
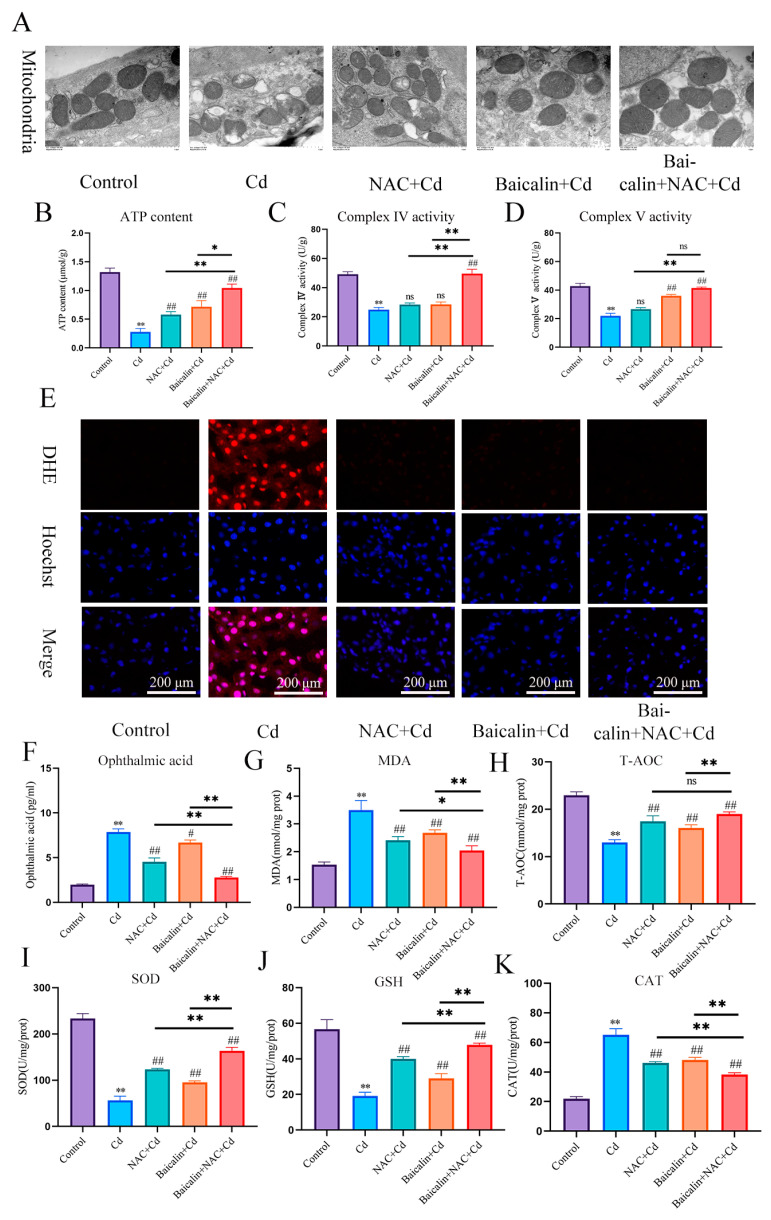
Baicalin and NAC combined to alleviate mitochondrial damage and oxidative stress. Mice in the NAC + Cd and Baicalin + Cd groups were given 20 mg/kg Baicalin and 10 mg/kg NAC, respectively, while being given 50 mg/L of Cd solution. The Transmission Electron Microscope (TEM) observed the ultrastructure of mitochondria (**A**) Scale bar = 1.0 μm ATP content (**B**) and mitochondrial respiratory complex IV (**C**) and V (**D**) activities were detected. DHE staining to observe the content of superoxide anions in tissues (**E**). Scale bar = 200 μm The contents of ophthalmic acid (**F**), MDA (**G**) and GSH (**J**), and the enzymatic activities of T-AOC (**H**), SOD (**I**), and CAT (**K**), were detected. The results were shown as the mean ± SD (n = 3). Compared with the control group, * *p* < 0.05, ** *p* < 0.01. Compared with the Cd group, # *p* < 0.05, ## *p* < 0.01. Compared with the Baicalin + NAC + Cd group, * *p* < 0.05, ** *p* < 0.01 ^ns^ *p* > 0.05.

**Figure 2:**
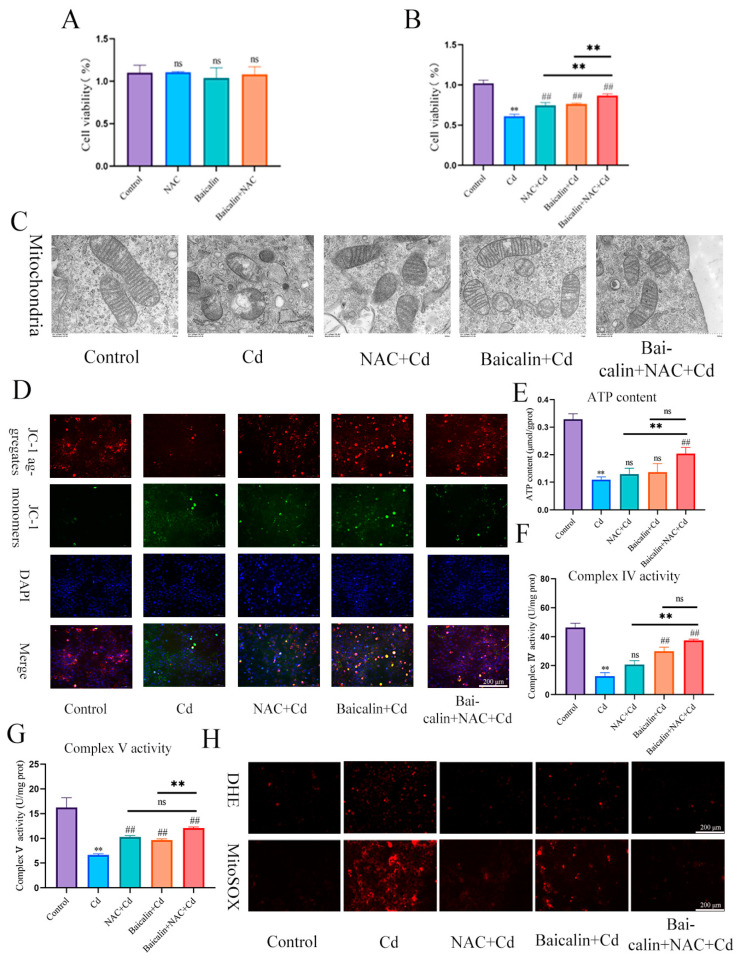
Baicalin and NAC in combination alleviates Cd-induced AML12 mitochondrial damage and ROS production. Cells were treated with 5 μM Cd, 10 μM Baicalin, and 20 μM NAC, alone or in combination, for 12 h. CCK8 detects cell viability (**A**,**B**), Transmission Electron Microscope (TEM) observed the ultrastructure of mitochondria (**C**). Scale bar = 0.5 μm. Detection of mitochondrial membrane potential by JC-1 staining (**D**) Scale bar = 200 μm. ATP content (**E**) and mitochondrial respiratory complex IV (**F**) and V (**G**) activity was detected. DHE and MitoSOX staining detect the content of superoxide anions and mitochondrial ROS (**H**). Scale bar = 200 μm. Results are shown as the mean ± SD (n = 3). Compared with the control group, ** *p* < 0.01. Compared with the Cd group, ^##^ *p* < 0.01. Compared with the Baicalin + NAC + Cd group, ^ns^ *p* > 0.05, ** *p* < 0.01.

In the present investigation, Cd intoxication disturbed the normal morphology of mitochondria, having broken the mitochondrial crest, and the mitochondrial membrane was ruptured as compared with the control group. However, NAC + Cd or Baicalin + Cd therapy when co-administrated substantially and notably overturned all the above-stated structural damages. Meanwhile, Baicalin + NAC + Cd therapy further restored the destruction of mitochondrial structure caused by Cd compared with the NAC + Cd and Baicalin + Cd groups (Figure 1A and Figure 2C).

We investigated the effects of baicalin and NAC on the activity of AML12 cells (the results are shown in Figure 2A,B). Baicalin and NAC alone or in combination did not affect cell activity, after co-treatment with Cd, the baicalin and NAC alleviated the Cd-induced decline in cell activity. ATP production is one of the main functions of mitochondria, so we further tested ATP content and mitochondrial respiratory chain complex enzyme activity, and the results showed that Cd significantly reduced ATP content and mitochondrial respiratory complex IV. and V activity. Compared with the Cd group, the NAC + Cd group increased the ATP content, but did not significantly increase the activity of mitochondrial respiratory complex IV. and V. Compared with the Cd group, the Baicalin + Cd group increased the content of ATP and the activity of mitochondrial respiratory complex V, but the effect on mitochondrial respiratory complex IV. was not obvious, and the Baicalin + NAC + Cd group increased the content of ATP and significantly increased the activity of mitochondrial respiratory chain IV compared with the NAC + Cd and Baicalin + Cd groups (Figure 1B–D and Figure 2E–G). The combination of baicalin and NAC showed better relief than baicalin alone. JC-1 detects mitochondrial membrane potential, and when mitochondrial membrane potential decreases, fluorescence changes from red to green. The results are shown in Figure 2D: Cd significantly reduces mitochondrial membrane potential, and baicalin or NAC alleviates Cd-induced decline in AML12 mitochondrial membrane potential. The combination of baicalin and NAC showed better relief than baicalin alone. The results of DHE staining showed that Cd significantly increased the intensity of DHE staining, baicalein or NAC alleviated the Cd-induced increase in DHE staining intensity, and the combination of baicalein and NAC further alleviated the increase in DHE staining (Figure 1E). In vitro, Cd significantly increased the fluorescence intensity of DHE and MitoSOX in AML12 cells. Baicalein or NAC alleviated Cd-induced increases in DHE and MitoSOX fluorescence intensity. The combination of baicalin and NAC showed better relief than baicalin alone (Figure 2H). Subsequently, we further tested antioxidant enzyme activity and oxidation product content. Cd significantly increased the content of MDA and ophthalmic acid and increased T-AOC and CAT, reducing SOD and GSH. Baicalein or NAC alleviated Cd-induced changes in antioxidant enzymes. The combination of baicalin and NAC showed more beneficial effects than baicalin alone (Figure 1F–K).

### 3.2. Combined Baicalin and NAC Reduce Lipid Accumulation

**Figure 3:**
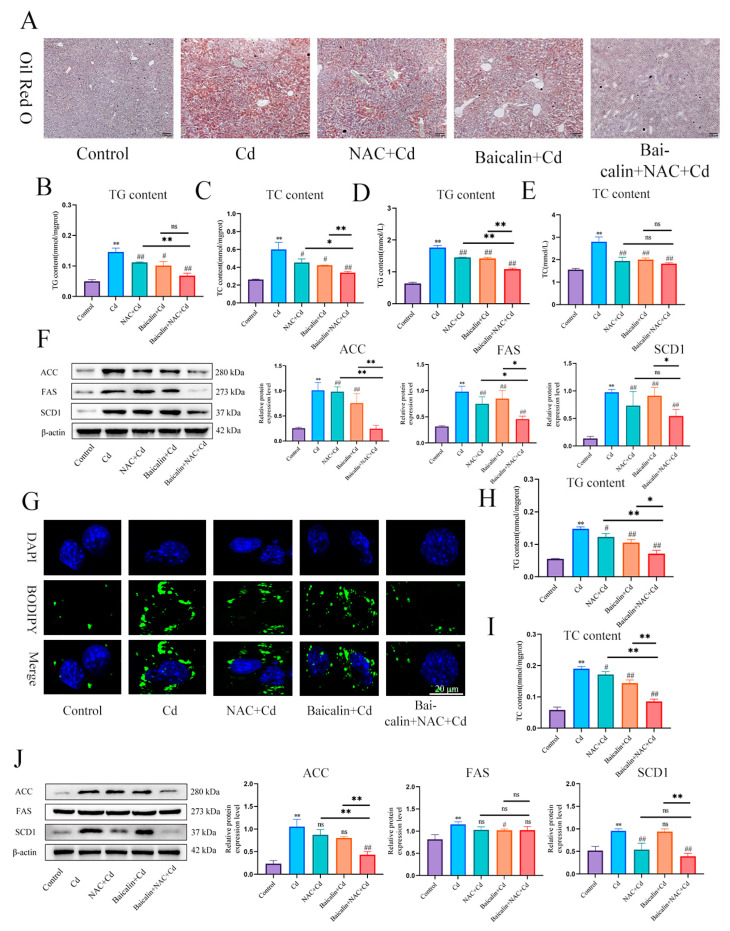
Baicalin and NAC in combination alleviates Cd-induced lipid accumulation. Oil red O staining to observe the content of neutral fat in tissues (**A**). Scale bar = 100 μm. Serum biochemistry to measure the levels of TG (**B**) and TC (**C**). TG (**D**) and TC (**E**) levels in the liver were also measured. Levels of ACC, FAS, and SCD1 were determined using Western blotting (**F**). Cells were treated with 5 μM Cd, 10 μM Baicalin, and 20 μM NAC alone or in combination, for 12 h. BODIPY staining detects AML12 intracellular lipids (**G**). TG (**H**) and TC (**I**) levels in the AML12 were also measured. Levels of ACC, FAS and SCD1 were determined using Western blotting (**J**). Results are shown as the mean ± SD (n = 3). Compared with the control group, * *p* < 0.05, ** *p* < 0.01. Compared with the Cd group, ^#^ *p* < 0.05, ^##^ *p* < 0.01. Compared with the Baicalin + NAC + Cd group * *p* < 0.05, ** *p* < 0.01, ^ns^ *p* > 0.05.

In order to detect the effects of baicalin and NAC on Cd-induced lipid accumulation, we used Oil red O (Figure 3A) and BODIPY staining (Figure 3G) to detect the content of lipids in liver and AML12 cells, respectively. Cd significantly increased the content of lipids, baicalin or NAC alleviated Cd-induced lipid elevations, and baicalin and NAC combined further alleviated the elevation of lipids. Triglycerides (TG) and total cholesterol (TC) kits detected the content of TG and TC in liver (Figure 3B–E) and AML12 cells (Figure 3H,I), baicalin or NAC alleviated Cd-induced elevation of TG and TC, and the combination of baicalin and NAC further alleviated the increase of TG and TC, but the combination of baicalin and NAC did not show better remission of TC compared with baicalin alone. Cd also significantly increased expression levels of ACC, FAS, and SCD1, and baicalin or NAC alleviated Cd-induced elevated levels of ACC, FAS, and SCD1. The combination of baicalin and NAC showed better relief than baicalin alone (Figure 3F,G).

### 3.3. Baicalin and NAC Reduce Lipid Accumulation by Promoting Lipophagy

**Figure 4:**
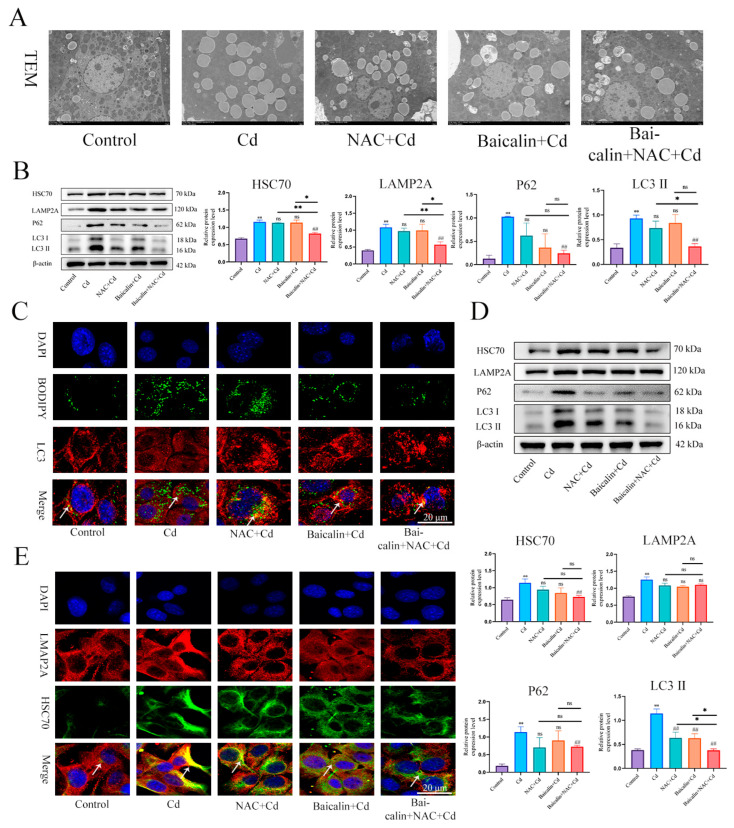
The combination of baicalin and NAC promotes lipophagy and alleviates Cd -induced lipid accumulation. TEM observes lipophagy in the liver (**A**). Scale bar = 2.0 μm. Levels of HSC70, LAMP2A, P62 and LC3 were determined using Western blotting (**B**). Immunofluorescence detection of lipid droplets and LC3 colocalization in AML12 cells (**C**). Levels of HSC70, LAMP2A, P62 and LC3 were determined using Western blotting in AML12 cells (**D**). Immunofluorescence detection of LAMP2A and HSC70 colocalization in AML12 cells (highlighted with white arrows) (**E**). Results are shown as the mean ± SD (n = 3). Compared with the control group, * *p* < 0.05, ** *p* < 0.01. Compared with the Cd group, ^##^ *p* < 0.01. Compared with the Baicalin + NAC + Cd group * *p* < 0.05, ** *p* < 0.01, ^ns^ *p* > 0.05.

TEM was used to observe lipophagy and showed that the Cd group significantly increased the number of intracellular lipid droplets compared to the control group, baicalin or NAC reduced the number of lipid droplets, and some lipid droplets were encapsulated by the membrane structure. The combination of baicalin and NAC further reduced the numbers of lipid droplets and increased the numbers of lipid droplets wrapped in the membrane structure (Figure 4A). BODIPY and LC3 indicate lipid droplets and autophagosomes, respectively. The results that are shown in Figure 4C indicate that, compared with the control group, the Cd group showed more lipid droplets, but the lipid droplets did not colocalize with LC3, while the combination of baicalin and NAC reduced the number of lipid droplets and promoted the colocalization of lipid droplets and LC3. Subsequently, we further examined the expression levels of HSC70, LAMP2, LC3, and P62 and showed found that Cd significantly increased the expression levels of HSC70, LAMP2, LC3, and P62, and the combination of baicalin and NAC reduced the expression levels of HSC70, LAMP2, LC3, and P62 (Figure 4B,D). Subsequently, we observed that the colocalization of HSC70 and LAMP2, the expression level of HSC70, and the colocalization of HSC70 and LAMP2 were significantly increased in the Cd group compared with the control group. Compared with the Cd group, the Baicalin + Cd group and NAC + Cd group reduced the expression level of HSC70 and LAMP2A, and the Baicalin + NAC + Cd group showed better remission effect than the Baicalin + Cd group and the NAC + Cd group (Figure 4E).

### 3.4. Combined Baicalin and NAC Promote Lipophagy by Inhibiting Chaperone-Mediated Autophagy

**Figure 5:**
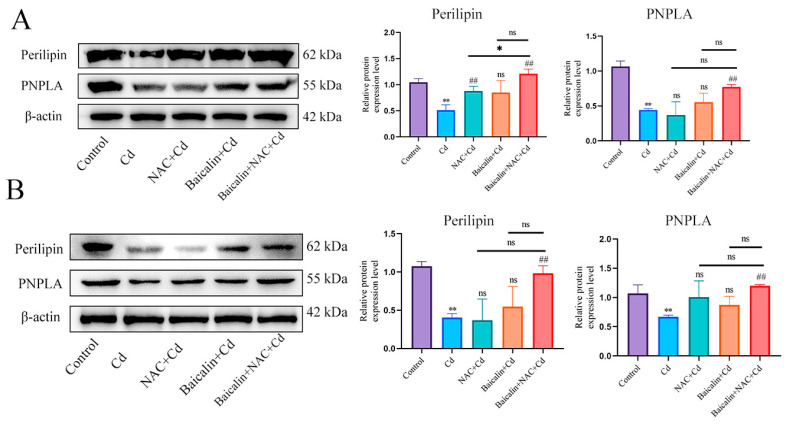
Combined use of baicalin and NAC restores Perilipin and PNPLA expression. Levels of Perilipin and PNPLA were determined using Western blotting (**A**). Levels of Perilipin and PNPLA were determined using Western blotting in AML12 cells (**B**). Results are shown as the mean ± SD (n = 3). Compared with the control group, * *p* < 0.05, ** *p* < 0.01. Compared with the Cd group, ^##^ *p* < 0.01. Compared with the Baicalin + NAC + Cd group ^ns^ *p* > 0.05, * *p* < 0.05, ** *p* < 0.01.

**Figure 6:**
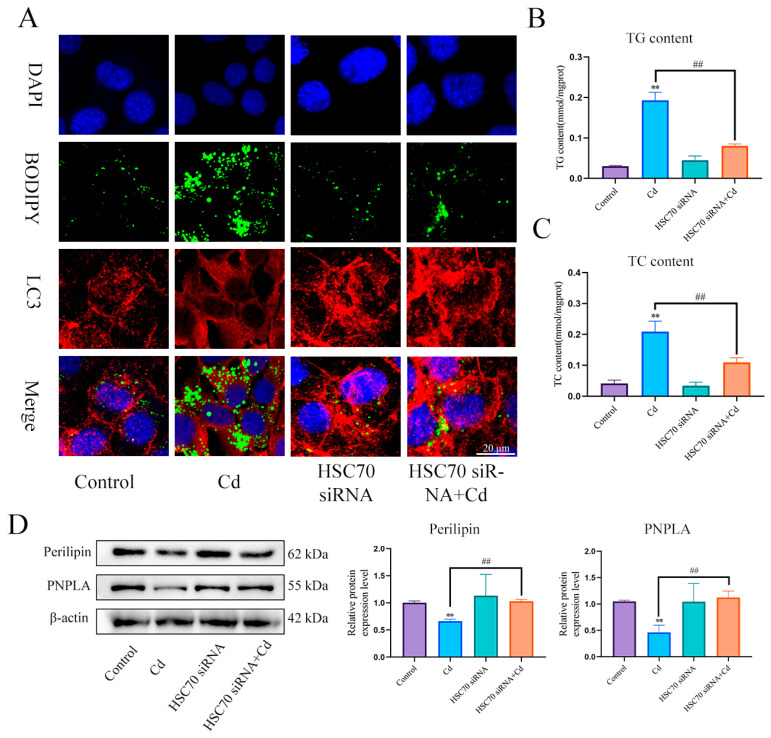
Inhibition of chaperone-mediated autophagy promotes lipophagy and reduces lipid accumulation. Based on treatment with HSC70 siRNA for 24 h, AML12 cells were treated with or without Cd for 12 h, Immunofluorescence detection of lipid droplets and LC3 colocalization in AML12 cells (**A**). TG (**B**) and TC (**C**) levels in the AML12 were also measured. Levels of Perilipin and PNPLA were determined using Western blotting in AML12 cells (**D**). Results are shown as the mean ± SD (n = 3). Compared with the control group, ** *p* < 0.01. Compared with the Cd group, ^##^ *p* < 0.01.

First, we examined the expression levels of Perilipin and PNPLA in liver (Figure 5A) and AML12 cells (Figure 5B). Cd significantly reduced the expression levels of Perilipin and PNPLA, and the combination of baicalin and NAC alleviated the reduction in Perilipin and PNPLA expression. To further validate the role of Cd on chaperone-mediated autophagy, we used a small interference technique (siRNA) to reduce the expression of HSC70. Based on treatment with HSC70 siRNA for 24 h, AML12 cells were treated with or without Cd for 12 h. Compared with the Cd group, the HSC70 siRNA + Cd group reduced the number of lipid droplets and promoted the colocalization of lipid droplets and LC3 (Figure 6A). The TG and TC results also showed that the HSC70 siRNA + Cd group reduced Cd-induced elevated TG and TC levels compared with the Cd group (Figure 6B,C) and alleviated the Cd-induced decrease in Perilipin and PNPLA expression levels (Figure 6D).

### 3.5. Mitochodrial ROS-Mediated Chaperone-Mediated Autophagy Inhibits Lipophagy

**Figure 7:**
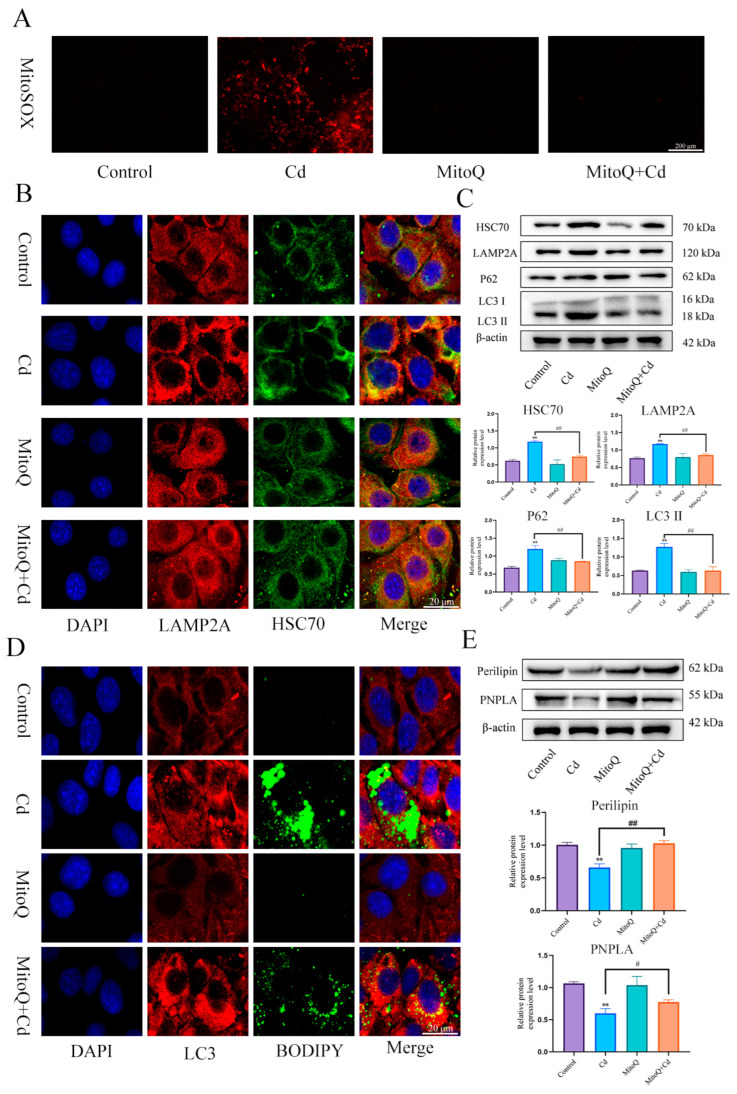
MitoQ alleviates Cd-induced chaperone-mediated autophagy and promotes lipophagy. We co-treated MitoQ with Cd for 12 h. MitoSOX detects mitochondrial ROS (**A**), Immunofluorescence detection of LAMP2A and HSC70 colocalization in AML12 cells (**B**). Levels of HSC70, LAMP2A, P62, and LC3 were determined using Western blotting in AML12 cells (**C**). Immunofluorescence detection of lipid droplets and LC3 colocalization in AML12 cells (**D**). Levels of Perilipin and PNPLA were determined using Western blotting in AML12 cells (**E**). Results are shown as the mean ± SD (n = 3). Compared with the control group, ** *p* < 0.01. Compared with the Cd group, ^#^ *p* < 0.05, ^##^ *p* < 0.01.

In order to detect the role of mitochondrial ROS in the baicalin and NAC promoting lipophagy, we co-treated MitoQ with Cd for 12 h, MitoQ significantly inhibited the production of Cd-induced mitochondrial ROS (Figure 7A). Then, we further detected the colocalization of HSC70 and LAMP2A, the MitoQ + Cd group had a significantly reduced the colocalization of HSC70 and LAMP2A compared with the Cd group (Figure 7B). HSC70, LAMP2, LC3, and P62 expression levels were tested, and MitoQ alleviated Cd-induced elevated levels of HSC70, LAMP2, LC3, and P62 (Figure 7C). We also examined the colocalization of lipid droplets and LC3, the MitoQ + Cd group promoted the colocalization of lipid droplets and LC3 compared to the Cd group (Figure 7D). We finally examined the expression levels of Perilipin and PNPLA, and MitoQ was found to alleviate the Cd-induced decline in Perilipin and PNPLA expression (Figure 7E).

### 3.6. PLD1 Regulates Choline to Alleviate Cadmium-Induced Mitochondrial Damage

**Figure 8:**
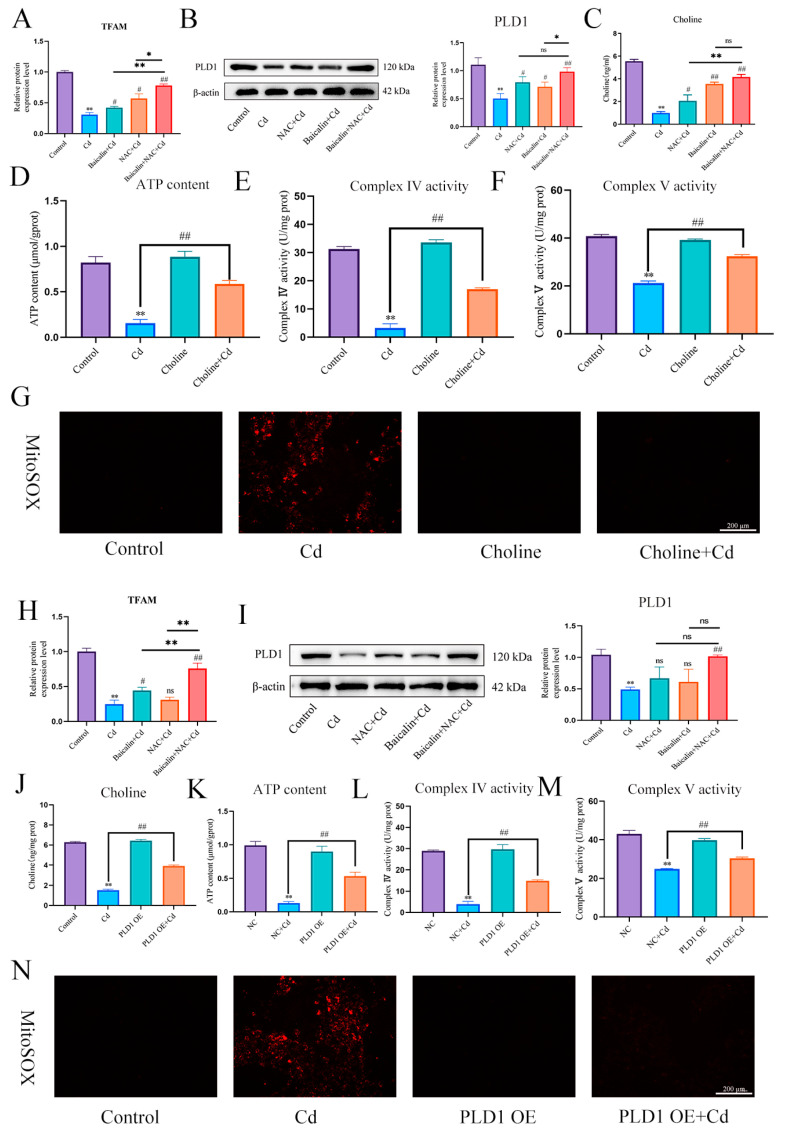
PLD1 regulates choline to alleviate Cd-induced mitochondrial damage. Elisa measured the amount of TFAM in mice (**A**) and AML12 cells (**H**). Levels of PLD1 were determined using Western blotting in mice (**B**) and AML12 cells (**I**). Elisa measures the amount of choline in mice (**C**). ATP content (**D**) and mitochondrial respiratory complex IV (**E**) and V (**F**) activity were detected. MitoSOX detects mitochondrial ROS (**G**). Based on treatment with the PLD1 OE plasmid for 24 h, AML12 cells were treated with or without Cd for 12 h, Elisa measures the amount of choline in AML12 (**J**). ATP content (**K**) and mitochondrial respiratory complex IV (**L**) and V (**M**) activity were detected. MitoSOX detects mitochondrial ROS (**N**). Results are shown as the mean ± SD (n = 3). Compared with the control group, * *p* < 0.05, ** *p* < 0.01. Compared with the Cd group, ^ns^ *p* > 0.05, ^#^ *p* < 0.05, ^##^ *p* < 0.01.

First, we found that Cd significantly reduced TFAM and PLD1 expression levels. The combination of baicalin and NAC alleviated the Cd-induced reduction in the expression levels of TFAM and PLD1 (Figure 8A,B,H,I). Elisa measured choline levels and showed that Cd significantly reduced choline levels, and the combination of baicalin and NAC alleviated the Cd-induced reduction in the choline levels (Figure 8C). Subsequently, in order to investigate the role of choline in the alleviation of Cd-induced mitochondrial damage by baicalin and NAC, we co-treated choline with Cd, choline alleviated Cd-induced decrease in ATP content and mitochondrial respiratory chain enzyme activity (Figure 8D–F), and MitoSOX staining also showed that choline reduced mitochondrial ROS (Figure 8G). When PLD1 was overexpressed, we found that the PLD1OE+Cd group alleviated Cd-induced choline (Figure 8J) deficiency and further alleviated Cd-induced mitochondrial damage compared to the Cd group (Figure 8K–M).

### 3.7. TFAM Regulates PLD1 to Stabilize Mitochondrial Structure and Function

**Figure 9:**
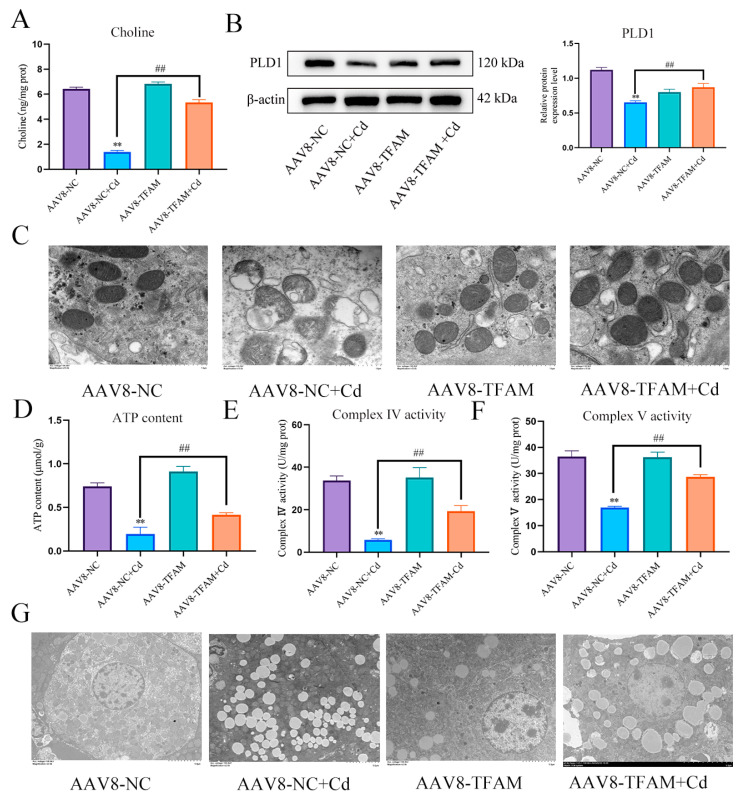
TFAM regulates PLD1 to alleviate Cd-induced mitochondrial damage and lipid accumulation. Elisa measures the amount of choline in mice (**A**). Levels of PLD1 were determined using Western blotting in mice (**B**). TEM observed the ultrastructure of mitochondria (**C**) Scale bar = 1.0 μm, ATP content (**D**) and mitochondrial respiratory complex IV (**E**) and V (**F**) activity were detected. TEM observes lipophagy in the liver (**G**) Scale bar = 5.0 μm. Results are shown as the mean ± SD (n = 3). Compared with the control group, ** *p* < 0.01. Compared with the Cd group, ^##^ *p* < 0.01.

TFAM is a mitochondrial transcription factor. The aim of this study is to investigate whether TFAM can mitigate Cd-induced mitochondrial damage by regulating PLD1. We measured the expression level of PLD1 and the content of choline, and the results showed that overexpression of TFAM increased the expression of PLD1 and the content of choline (Figure 9A,B). TEM observed mitochondrial structure, compared with the Cd group, and the AAV8-TFAM + Cd group alleviated the destruction of mitochondrial membrane structure (Figure 9C). ATP content and mitochondrial respiratory chain enzyme activity also showed the same trend, i.e., the overexpression of TFAM alleviated Cd-induced decrease in ATP content and decreased mitochondrial respiratory chain enzyme activity (Figure 9D–F). TEM was used to observe lipid droplets in tissues, and the results showed that overexpression of TFAM significantly reduced the number of lipid droplets in the liver and promoted lipophagy (Figure 9G).

### 3.8. Loss of TFAM Promotes Chaperone-Mediated Autophagy and Thus Inhibits Lipophagy

**Figure 10:**
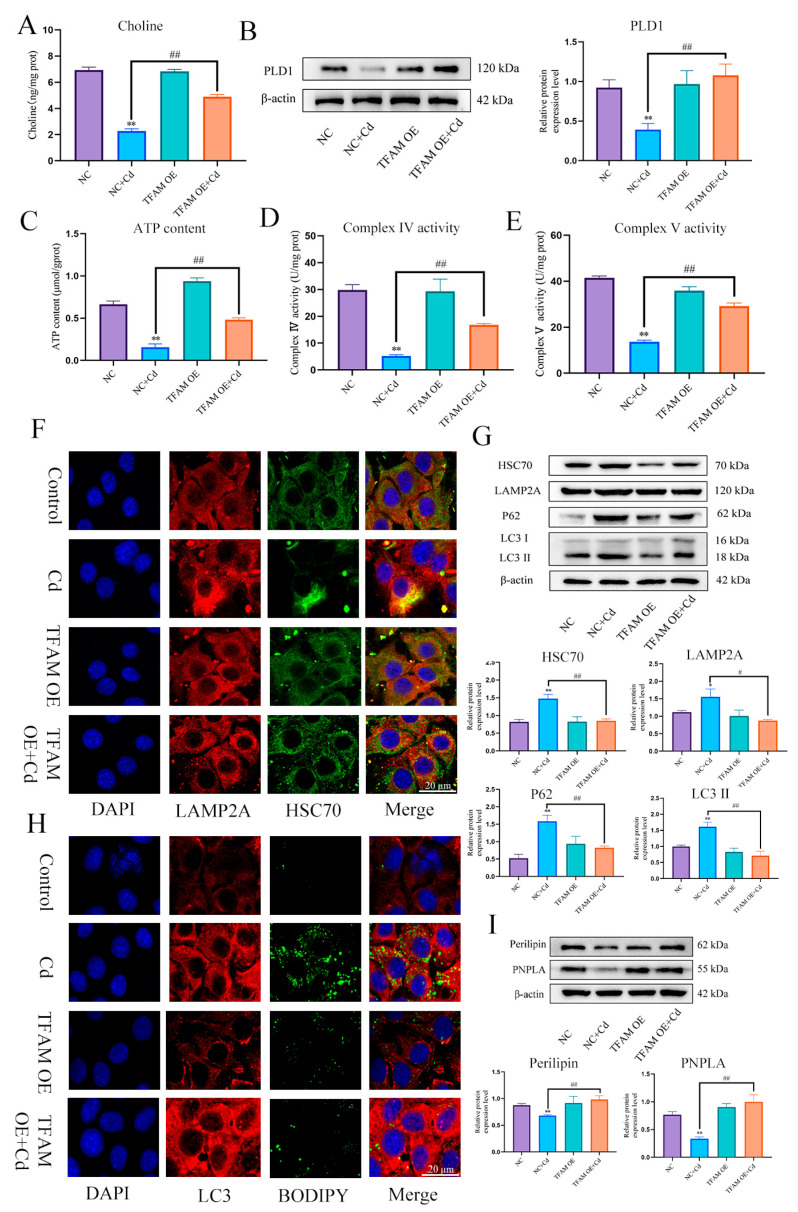
TFAM reduces chaperone-mediated autophagy and promotes lipophagy to alleviate cadmium-induced lipid accumulation. Elisa measures the amount of choline in mice (**A**). Levels of PLD1 were determined using Western blotting in mice (**B**). ATP content (**C**) and mitochondrial respiratory complex IV (**D**) and V (**E**) activity was detected. Immunofluorescence detection of LAMP2A and HSC70 colocalization in AML12 cells (**F**). Levels of HSC70, LAMP2A, P62 and LC3 were determined using Western blotting in AML12 cells (**G**). Immunofluorescence detection of lipid droplets and LC3 colocalization in AML12 cells (**H**). Levels of Perilipin and PNPLA were determined using Western blotting in AML12 cells (**I**). Results are shown as the mean ± SD (n = 3). Compared with the control group, * *p* < 0.05, ** *p* < 0.01. Compared with the Cd group, ^#^ *p* < 0.05, ^##^ *p* < 0.01.

In order to further verify the role and mechanism of TFAM, based on treatment with TFAM OE for 24 h, AML12 cells were treated with or without Cd for 12 h, compared with Cd group, the TFAM OE + Cd group increased the expression level of PLD1 and choline content (Figure 10A,B), and increased the content of ATP and the enzyme activity of mitochondrial respiratory chain (Figure 10C–E). The colocalization of HSC70 and LAMP2A was also detected, and the results showed that overexpression of TFAM reduced the colocalization of HSC70 and LAMP2A (Figure 10F). We also examined the expression levels of HSC70, LAMP2, LC3, and P62. The expression levels of HSC70, LAMP2, LC3, and P62 were reduced in the TFAM OE + Cd group compared with the Cd group (Figure 10G). Finally, we observed the colocalization of lipid droplets and LC3 and the expression levels of Perilipin and PNPLA. The overexpression of TFAM increased the colocalization of lipid droplets and LC3 and the expression levels of Perilipin and PNPLA (Figure 10H,I).

## 4. Discussion

Traditional Chinese medicine has demonstrated effective therapeutic effects in the context of various diseases through the collaboration of Chinese herbal medicines various combinations. Over the last decade, the utilization of multiple Chinese herbal medicines has influenced the development of modern medicine, moving which has moved from a single-drug treatment model to the application of combination therapies [24]. Recent evidence suggests that combination therapy can offer increased therapeutic benefits and effectiveness in relation to complex conditions such as Acquired Immune Deficiency Syndrome (AIDS), cancer, atherosclerosis, and diabetes, which were previously challenging to treat [25,26,27]. It is essential to note that this therapy approach has shown promising results in numerous studies and holds significant potential for further research and development. Baicalin, which originates from the roots of scutellaria, is a type of flavonoid and is the primary active substance belonging to flavonoids that safeguarding mitochondria from the external environment [28]. NAC, an antioxidant, demonstrates excellent therapeutic efficacy in a range of liver, and kidney diseases, and can be utilized synergistically as an adjunct to treat diseases [29]. It is uncertain whether the combination therapy of baicalin and NAC can mitigate Cd-induced lipid accumulation. Nevertheless, in our study, discovered that combining baicalin and NAC did alleviate Cd-induced lipid accumulation.

Mitochondria, dual-membrane organelles catalyzing oxidative phosphorylation for adenosine triphosphate (ATP) are pivotal for energy homeostasis, apoptosis, metabolic signaling, intracellular calcium balance, and lipid synthesis [30]. They are deeply linked to numerous disorders, including neurodegeneration, cancer, cardiac rhythm disorders, obesity, and diabetes. [31]. The essential protein component TFAM plays an instrumental role in mitochondrial DNA (mtDNA) maintenance and is indispensable for in preserving its structure and facilitating transcription and replication [32]. While the global knockout of TFAM in embryonic development proves lethal, the targeted disruption thereof within tissues replicates mitochondrial disease manifestations found across various patient populations [33,34,35]. Reduced TFAM and mtDNA levels lead to decreased mitochondrial activity, and the TFAM-mediated regulation of various cancers’ carcinogenicity may occur through the disruption of LC3 II-mediated autophagy [36]. Studies have shown that a variety of heavy metals, such as Cd, arsenic, and mercury, can disrupt mitochondrial function and increase mitochondrial ROS production [37], and Zhao et al. found that circRNA SCAR targeting mitochondrial localization alleviated NASH by reducing mROS production [38]. In this study, we investigated the effects of baicalein and NAC on Cd-induced mitochondrial damage. Our findings indicate that Cd significantly disrupts the membrane structure and function of mitochondria, increases mitochondrial ROS production, and promotes lipid accumulation. The combination of baicalin and NAC synergistically alleviates Cd-induced mitochondrial damage and lipid accumulation. Interestingly, we observed choline deletion at the same time, and baicalin and NAC alleviated Cd-induced choline deficiency. Choline is an important component of mitochondrial membranes [39]. Dietary deficiency limitations of choline disrupt the function of mitochondrial respiratory chain complex 1 and promote the production of H_2_O_2_ [40]. Therefore, it is speculated that choline could be a potential target for the combination of baicalin and NAC to alleviate Cd-induced mitochondrial damage and lipid accumulation. Subsequently, choline was co-treated with Cd, and it was observed that choline alleviated Cd-induced mitochondrial damage and inhibited lipid accumulation in hepatocytes. The liver can use phospholipase to break down phospholipids to phosphatidic acid and choline, in addition to dietary choline intake [41]. This study investigates the effects of Cd on PLD1 expression and choline loss, as well as the potential protective effects of baicalin and NAC. Results show that Cd significantly reduced PLD1 expression, which was restored by baicalin and NAC combination. Overexpression of PLD1 reversed Cd-induced choline loss and mitochondrial damage. Additionally, the baicalin and NAC combination increased the expression of TFAM. To determine whether TFAM can regulate PLD1, we established an in vivo and in vitro model of TFAM overexpression. The results show that TFAM overexpression can inhibit Cd-induced choline loss by regulating PLD1 while stabilizing the structure and function of mitochondria. TFAM acts as an upstream signal for baicalin and NAC to alleviate Cd-induced mitochondrial damage and regulates choline metabolism through PLD1, thereby alleviating mitochondrial damage.

Autophagy is an essential catabolic system found in eukaryotes [42]. It can be classified into three types: macroautophagy, microautophagy, and chaperone-mediated autophagy [43]. Scholars have identified a significant role for autophagy in the progression of non-alcoholic fatty liver disease (NAFLD). Suppressed autophagy results in excessive lipid aggregation, subsequently triggering steatosis, steatohepatitis, and carcinogenesis [44]. In rodent livers, As2O3, a potent inhibitor of autophagy, stimulates NLRP3 inflammasomes, intensifying NASH [45]. Cd disrupts autophagy flow by inhibiting the fusion of autophagosomes and lysosomes [46]. This impedes cholesterol distribution and amplifies hyperlipidemia via disruption of the autophagy–lysosomal pathway [47]. In this study, we found that Cd inhibited lipophagy, which was consistent with previous studies, and that baicalin and NAC combined to alleviate cadmium’s inhibition of lipophagy. Interestingly, we also found that Cd promoted CMA, and the combination of baicalin and NAC alleviated the increase in the level of CMA. Studies have shown that CMA promotes lipophagy by degrading Perilipin [48,49]. This contradicts this study. At the same time, we discovered that Cd caused the loss of PNPLA. PNPLA has been reported as an LC3 receptor that promotes the formation of autophagosome membranes [50]. And after the inhibition of CMA, lipophagy levels were restored. We then thus infer that Cd induces CMA, which degrades PNPLA at the same time as Perilipin. This leads to the inhibition of lipophagy, while baicalin and NAC can inhibit Cd-induced CMA, thereby promoting lipophagy and reducing lipid accumulation. Autophagy inhibition in NAFLD is often accompanied by oxidative stress [51,52]. To further determine how Cd induces CMA and the role of baicalin and NAC in this process. We co-treated with Cd using MitoQ and showed that MitoQ inhibited Cd-induced CMA and restored lipophagy. This indicates that mitochondrial ROS is a prerequisite for Cd-induced CMA. The combination of baicalin and NAC can promote lipophagy by inhibiting the production of mitochondrial ROS.

## 5. Conclusions

In conclusion, these findings suggest that the combination of baicalin and NAC could be a promising approach to reducing Cd-induced lipid accumulation. In this study, we discovered a novel pathway by which Cd induces lipid accumulation by increasing mitochondrial ROS-mediated CMA and disrupting lipophagy, thereby increasing lipid accumulation. We also found that mitochondria are one of the targets of baicalin and NAC combination, which regulates choline metabolism through TFAM-PLD1 to stabilize mitochondrial structure and function, inhibit the production of mitochondrial ROS, reduce the level of CMA, and promote lipophagy, thereby alleviating Cd-induced lipid accumulation.

## Data Availability

The datasets used and/or analyzed during the current study are available from the corresponding author on reasonable request.
